# A Simple PCR Method for Rapid Genotype Analysis of the *TH-MYCN* Transgenic Mouse

**DOI:** 10.1371/journal.pone.0006902

**Published:** 2009-09-04

**Authors:** Seiki Haraguchi, Akira Nakagawara

**Affiliations:** 1 Laboratory of Embryonic and Developmental Genetics, Chiba Cancer Center Research Institute, Chuo-ku, Chiba, Japan; 2 Division of Biochemistry and Innovative Cancer Therapeutics, Chiba Cancer Center Research Institute, Chuo-ku, Chiba, Japan; University of Arkansas for Medical Sciences, United States of America

## Abstract

**Background:**

The *TH-MYCN* transgenic mouse is the most widely used murine model of human neuroblastoma, in which a human *MYCN* oncogene is targeted to neuroectodermal cells of developing mice under the influence of the rat *tyrosine hydroxylase* promoter. So far, homozygous transgenic mice have been identified by either Southern blot or quantitative real-time PCR.

**Principal Findings:**

To establish a simple and reliable genotyping method by conventional PCR, we confirmed the integration of the transgene in the *TH-MYCN* transgenic mouse by Southern blot and inverse PCR analyses. Our results showed that either five or six copies were found to be inserted in a head-to-tail tandem configuration at a single locus. The *MYCN* transgene/host DNA junction was sequenced and the integration site was identified at chromosome 18qE4. Finally, we succeeded in designing rapid, simple and reliable genotyping method by common PCR using primers flanking the integrated *TH-MYCN* transgene.

**Conclusion:**

We established a simple and reliable genotyping PCR method for determining the integration site of the *TH-MYCN* transgene that enables all possible genotypes to be distinguished within several hours. *TH-MYCN* mice are excellent model for human neuroblastoma study, thus our results will largely be useful for facilitating the pace of neuroblastoma study, including in the study of the tumourigenic process, and in the development of therapies to treat patients suffering from neuroblastoma.

## Introduction

Amplification of *MYCN* is a genetic abnormality found in approximately one third of all human neuroblastomas and closely associated with advanced stage and poor outcome [1], [2]. A murine model of neuroblastoma has been established by targeted expression of the human *MYCN* oncogene in neuroectodermal cells under the control of rat *tyrosine hydroxylase* promoter (*TH-MYCN*) [3]. Use of transgenic mice expressing the human *MYCN* oncogene has provided definitive evidence for the role of MYCN in neuroblastoma tumorigenesis, recapitulating several biological and histological aspects of clinical neuroblastomas [3], [4], [5].

There are circumstances where both hemizygous and homozygous transgenic mice are necessary to explore the role of MYCN in neuroblastomas. So far, homozygous transgenic mice have been identified by either Southern blot [3] or quantitative real-time PCR [6]. Quantitative real-time PCR is dependent on the quantification of differential amounts of transgene DNA between hemizygous and homozygous transgenic mice, thus, all procedures should be carried out meticulously with particular attention to the quality of DNA preparation [6]. Additionally, reliable results of genotyping may take several days to obtain.

Inverse PCR (IPCR) has numerous applications in molecular biology including amplification and identification of genomic inserts [7]. IPCR is generally used if only one internal sequence of the target DNA is known. It is therefore especially useful in identifying flanking DNA sequences of genomic inserts. If one or more of the *TH-MYCN* transgene/host DNA junctions have been sequenced, it is convenient to design a PCR to distinguish homozygotes from hemizygotes. With the aim of facilitating genotype analysis of *TH-MYCN* transgenic mice, we analyzed the arrangement of integrated DNA in transgenic mice by Southern blot and inverse IPCR analyses.

In this study, we were successful to identify the transgene locus, in which head-to-tail tandem repeats were integrated at a single insertion site at chromosome locus 18qE4. Using primers flanking the integrated *TH-MYCN* transgene, rapid and reliable genotyping was achieved by conventional PCR.

## Materials and Methods

### Mice

129X1/SvJ strain of *TH-MYCN* transgenic mice [3] were backcrossed with 129^+*Ter*^/SvJ strain (CLEA, Japan) in our animal facility. In this study, both substrains were examined. All of the mice were maintained and used in accordance with guidelines issued by the Institutional Animal Care and Use Committee.

### Genomic DNA preparation

For genotyping PCR analysis [8], 1–2 mm sections of tail tip were dissolved in 0.1 ml of 50 mM Tris (pH 8.0), 100 mM EDTA, 0.5% SDS, and 0.5 mg/ml proteinase K (Roche) solution at 55°C for at least one hour with vigorous shaking. The DNA was purified by phenol/chloroform extraction followed by ethanol precipitation and then dissolved in 0.5 ml of TE solution. For Southern blot and inverse PCR analyses, genomic DNA was prepared from digested liver using DNA Isolation Kit for Cells and Tissues (Roche).

### Southern blot

**Figure 1:**
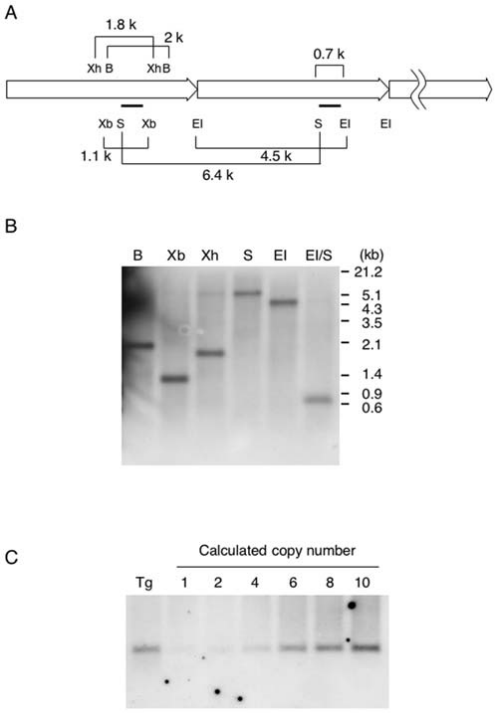
Tandem copies of the transgene are arrayed head-to-tail at a single insert locus. (A) Schematic diagram of restriction enzyme sites and the sizes expected by Southern blot analysis. Bold lines show the position of the probe. B, *BamHI*; EI, *EcoRI*; S, *SalI*; Xb, *XbaI*; Xh, *XhoI*. (B) Southern blot analysis. Genomic DNA from *MYCN* ^Tg/+^ mouse was digested with indicated restriction enzymes. Note that all results are consistent with the expected size shown in (A). (C) Quantification of transgene copy number. Genomic DNA (5 µg per lane) from *MYCN* ^Tg/+^ and wild type mice were digested with *EcoRI* and *SalI*. Copy standards were prepared by mixing wild type DNA with a known amount of DNA and subjected to Southern blot analysis. *MYCN* ^Tg/+^ mouse has about 5–6 copies of the transgene.

**Figure 2:**
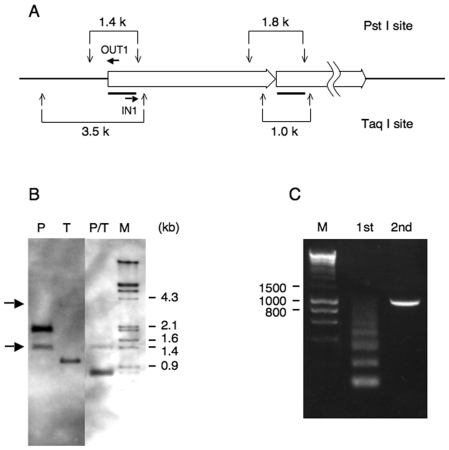
IPCR after *PstI* restriction enzyme digestion. (A) Restriction enzyme map of *PstI* and *TaqI*. *PstI* and *TaqI* sites are located approximately 0.7 kb and 2.5 kb up-stream of the transgene, respectively. The bold lines indicate location of the 0.7 kb probe. Primers (OUT1 and IN1) were used for the 2nd IPCR. (B) Southern blot analysis. In addition to the expected size, an additional band (indicated by arrows) was detected by *PstI* (1.4 kb) and *TaqI* (3.5 kb) digestion. (C) *PstI* digested DNA fragment (1.4 kb) was eluted from the gel, purified and IPCR was carried out. The expected size (1 kb) of the 2nd PCR product was obtained.

DNA was thoroughly digested with the appropriate restriction endonucleases. Digested DNA was separated by 1.0% agarose gel and alkaline transferred to a positively charged nylon membrane (Roche). DIG-labeled DNA probes were used for Southern blot analysis (Fig. 1A, 2A) and all procedures for the DIG application system (Roche) were performed according to the manufacturer's recommendations.

### Inverse PCR

Genomic DNA digested with *PstI* restriction enzyme was separated by agarose gel electrophoresis with molecular weight marker and the region corresponding to 1.4 kb was cut out. DNA was eluted using QIAquick Gel Extraction Kit (Qiagen) and self-ligated with T4 DNA ligase (New England BioLabs) at 16°C over night. After ethanol precipitation, PCR was carried out using Advantage cDNA polymerase mix (Clontech) with the following conditions: 95°C 60 s (1 cycle); 95°C 20 s, 68°C 180 s (35 cycles). First PCR was performed using primer sets:

OUT2, 5′-ACAGAGACACATGACCACACATATATGAGGAC-3′

IN2, 5′-GTCATGATGCTGGTTGAAAGTGGCCTTTG-3′

The first PCR product was used for a second nested PCR using the following primer sets:

OUT1, 5′-TTGGCACACACAAATGTATATACACAATGG-3′

IN1, 5′-AAGACCAAGGATCAGGACACCCCCTAGT-3′

Amplified fragments were separated by 1.5% agarose gel electrophoresis, purified and finally sequenced using OUT1 and IN1 primers respectively.

### Genotyping

PCR was carried out using rTaq (Takara) with the following conditions: 95°C 60 s (1 cycle); 95°C 20 s, 58°C 30 s, 72°C 40 s (34 cycles) (N008/N009 and Chr18F1/Chr18R2/OUT1), or using Prime STAR Max (Takara) with the following conditions: 98°C 30 s (1 cycle); 98°C 10 s, 68°C 40 s (34 cycles) (Chr18F5/Chr18R2/hMYCNF). Primers used for the analysis were as follows, in which OUT1 is listed above:

N008, 5′-TGGAAAGCTTCTTATTGGTAGAAACAA-3′

N009, 5′-AGGGATCCTTTCCGCCCCGTTCGTTTTAA-3′

hMYCNF, 5′-TCCAGCGAGCTGATCCTCAAACGATGCC-3′

Chr18F1, 5′-ACTAATTCTCCTCTCTCTGCCAGTATTTGC-3′

Chr18R2, 5′-TGCCTTATCCAAAATATAAATGCCCAGCAG-3′

Chr18F5, 5′-ATCTGACATTAAACTTGTGGAGGCCTAGAC-3′

## Results and Discussion

We first determined the integration patterns of transgenes. The *TH-MYCN* transgenic vector consists of about 4 kb of rat *TH* promoter, 0.7 kb of rabbit *β-globin* intron, 1.7 kb of human *MYCN* cDNA, and 0.1 kb of herpes simplex virus thymidine kinase gene terminator [3]. Unfortunately, we do not have detailed information about the restriction enzyme sites or sequence of the vector. In spite of this, when multiple copies are present, they are usually found at a single chromosomal locus [8]. If multiple copies are integrated in head-to-tail tandem configuration into a single locus of genome, Southern blot analysis reveals the expected size after digestion with the appropriate restriction enzyme as shown in Fig. 1A. The probe used for this experiment corresponds to a 0.7 kb region of rabbit *β-globin* intron because both rat *TH* promoter and human *MYCN* gene have high homology compared with their mouse orthologs (more than 80%, data not shown). Each DNA fragment digested with *BamHI* (2 kb), *XbaI* (1.1 kb), *XhoI* (1.8 kb), *SalI* (6.4 kb), *EcoRI* (4.5 kb), and *EcoRI/SalI* (0.7 kb) was consistent with the predicted size (Fig. 1A, 1B). This result showed that more than 2 copies of transgenes are integrated in head-to-tail tandem arrays at a single chromosomal locus. We next examined approximate copy numbers using copy standards. Weiss et al. estimated that one lineage of *TH-MYCN* transgenic mice had about 4 copies of the transgene by Southern blot analysis [3]. Similar to this, our result showed that the estimated copy number was five to six (Fig. 1C). The widely used murine model for neuroblastoma is *TH-MYCN* transgenic mouse established by Weiss et al. [3], which is currently provided by National Cancer Institute (NCI). The origin of *TH-MYCN* transgenic mice in our facility is derived from NCI. Thus, the *TH-MYCN* transgenic mice we used in this study are the same lineage commonly used in neuroblastoma studies.

IPCR is a method for the rapid in vitro amplification of DNA sequences that flank a region of known sequence. The individual digested restriction fragments are self-ligated with a T4 DNA ligase and the resultant circular DNA is then used as a template for PCR [7]. To perform IPCR, we first checked whether there are restriction enzyme sites in the sequences adjacent to the junction. DNA was digested with several combinations of restriction enzymes (data not shown) and Southern blot analysis was performed using a 0.7 kb probe spanning the 5′ region of the rat *TH* promoter (Fig. 2A). As expected, an additional clear band was obtained by digestion with either *PstI* (1.4 kb) or *TaqI* (3.5 kb) restriction enzymes (Fig. 2B). In addition, the 1.4 kb band was also confirmed by *PstI* and *TaqI* double digestion (Fig. 2B). This result suggested that both *PstI* and *TaqI* restriction enzyme sites are mapped to approximately 0.7 kb and 2.5 kb up-stream of the *TH* promoter, respectively (Fig. 2B shows as 1.4 kb and 3.5 kb, respectively). The 1.4 kb of DNA fragment was eluted from the gel, purified and self-ligated in preparation for IPCR. The 2nd PCR product obtained as an expected size of 1 kb (Fig. 2C) was sequenced and referred to genomic BLAST databases.

**Figure 3:**
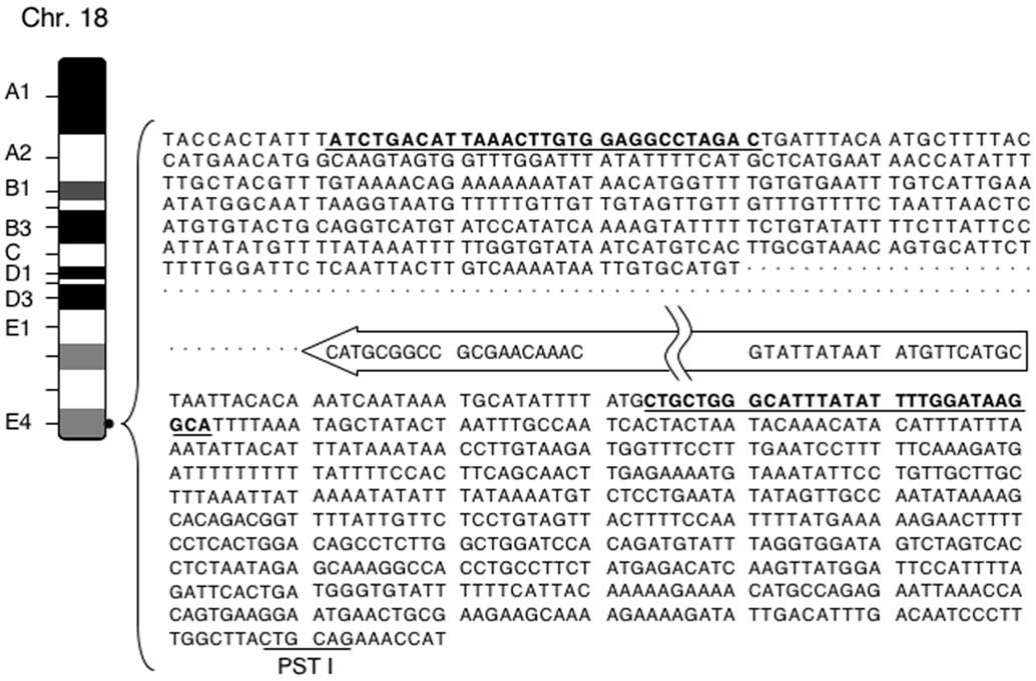
*TH-MYCN* transgene is integrated in a Chr18qE4 locus. The sequence of the transgene/host DNA junctions are shown. Bold letters with underlines indicate the primers (Chr18F5 and Chr18R2) using for genotyping. *PstI* site is located 610 bp up-stream of the transgene. Dotted lines indicate 1,063 bp of the genomic DNA deletion. It should be noted that the deletion was shown down-stream of the transgene for convenience (see results and discussion).

As shown in Fig. 3, sequencing demonstrated insertion of *MYCN* transgene into the E4 locus of chromosome 18q, which is included in the clone RP24-112L21 (GenBank accession number: AC118642). It has been reported that the amplification on chromosome 18 in *TH-MYCN* transgenic mice was observed by CGH analysis and suggested that distal chromosome 18 would be the site of *TH-MYCN* transgene integration [9]. Consistent with the previous data, our result proved this by sequencing. Although the reference sequence is derived from C57BL/6, our sequence data from both junctions completely matched, additionally *PstI* and *TaqI* restriction sites were also confirmed 0.6 kb (Fig. 2B) and 2.1 kb (data not shown) up-stream of the *TH* promoter. Moreover, primers used in this study worked well for genotyping (see below). Interestingly, a 1 kb deletion of the host genome was observed at the junction site. Although it is unknown which side of the transgene contains this deletion, the genomic deletion is shown by a dotted line at the down-stream of the transgene (Figs. 3). The sequences of both ends of the transgene were retained (Fig. 3).

**Figure 4:**
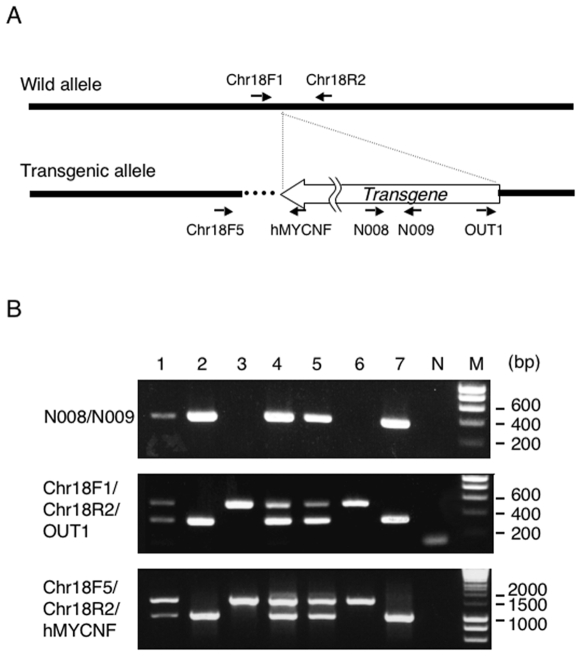
Genotyping PCR using flanking primer sets. (A) The position of primers. Dotted line shows genomic deletion (see results and discussion). (B) PCR with the primers N008/N009 identifies either transgenic or non-transgenic mice, in which the product is detected as 400 bp band. On the other hand, multiple PCR with three primer sets clearly distinguish wild-type (single larger band), homozygous (single smaller band) and hemizygous (both bands) DNA. The expected size of PCR products are 500 bp and 295 bp with Chr18F1/Chr18R2/OUT1, and 1.5 kb and 1 kb with Chr18F5/R2/R3.

Finally, we performed genotyping PCR using flanking primer sets (Fig. 4A). Fig. 4B shows the results of genotyping of pups resulting from inter cross mating. Although using primer set N008/N009, it is possible to distinguish between transgenic and non-transgenic mice, it is still unknown whether those transgenic mice are hemizygotes or homozygotes (Fig. 4A and B upper panel). However, multiple PCR with 3 primers, Chr18F1/Chr18R2/OUT1 and Chr18F5/Chr18R2/hMYCNF, recognizing either the 5′ or 3′ regions of the transgene enables all possible genotypes to be distinguished by the different size of the products. The multiple PCR clearly showed wild type (single larger band, #3 and #6), hemizygous (two bands, #1, #4 and #5), and homozygous (single smaller band, #2 and #7) transgenic mice (Fig. 4B middle and lower panels).

In summary, we established a simple and reliable genotyping PCR method for determining the integration site of the *MYCN* transgene. Unlike quantitative real-time PCR, conventional PCR can be performed with general PCR equipment and materials. Moreover, easily and immediately determining an accurate genotype is necessary for facilitating the pace of neuroblastoma study. Thus, this conventional PCR genotyping method should be widely used for neuroblastoma study.
